# Network Pharmacology and Molecular Docking Analysis on Molecular Targets and Mechanisms of Buyang Huanwu Decoction in the Treatment of Ischemic Stroke

**DOI:** 10.1155/2021/8815447

**Published:** 2021-02-26

**Authors:** Qiang Gao, Danfeng Tian, Zhenyun Han, Jingfeng Lin, Ze Chang, Dandan Zhang, Dayong Ma

**Affiliations:** ^1^Beijing University of Chinese Medicine, Beijing 100029, China; ^2^Shenzhen Hospital of Beijing University of Chinese Medicine (Longgang), Shenzhen 518172, China; ^3^Neurology Department of Dongzhimen Hospital, Beijing University of Chinese Medicine, Beijing 100700, China

## Abstract

**Methods:**

The bioactive components and potential targets of BHD were screened by TCMSP, BATMAN-TCM, ETCM, and SymMap databases. Besides, compounds that failed to find the targets from the above databases were predicted through STITCH, SwissTargetPrediction, and SEA. Moreover, six databases were searched to mine targets of IS. The intersection targets were obtained and analyzed by GO and KEGG enrichment. Furthermore, BHD-IS PPI network, compound-target network, and herb-target-pathway network were constructed by Cytoscape 3.6.0. Finally, AutoDock was used for molecular docking verification.

**Results:**

A total of 235 putative targets were obtained from 59 active compounds in BHD. Among them, 62 targets were related to IS. PPI network showed that the top ten key targets were IL6, TNF, VEGFA, AKT1, etc. The enrichment analysis demonstrated candidate BHD targets were more frequently involved in TNF, PI3K-Akt, and NF-kappa B signaling pathway. Network topology analysis showed that *Radix Astragali* was the main herb in BHD, and the key components were quercetin, beta-sitosterol, kaempferol, stigmasterol, etc. The results of molecular docking showed the active components in BHD had a good binding ability with the key targets.

**Conclusions:**

Our study demonstrated that BHD exerted the effect of treating IS by regulating multitargets and multichannels with multicomponents through the method of network pharmacology and molecular docking.

## 1. Introduction

The incidence of ischemic stroke (IS) is particularly high, and survivors have more or less neurological function deficits, which brings about a large burden on society and patients' families. With high mortality and morbidity rate, stroke has been the third most common cause of death following by coronary heart disease and cancer in the world [1]. IS accounts for 70%–80% of all stroke, which is the most common type of stroke in clinic [2]. At present, thrombolysis has been considered as the fastest and most effective treatment for IS, but the clinical effect of thrombolysis therapy is limited due to strict indications, a short time window, high risk of bleeding, and reperfusion injury [3]. A large number of experimental studies and clinical observations have confirmed that Chinese medicine has unique advantages in treating IS. Various empirical prescriptions, single drugs, and active ingredient extracts have shown clear neuroprotective effects on IS [4–6].

Buyang Huanwu decoction (BHD) is a classical prescription for the treatment of IS. This prescription is mainly composed of Huangqi (*Radix Astragali*), Honghua (*Carthami flos*), Taoren (*Persicae Semen*), Chishao (*Radix Paeoniae Rubra*), Danggui (*Angelicae sinensis Radix*), Chuangxiong (*Chuanxiong Rhizoma*), and Dilong (*Pheretima*). Among them, *Radix Astragali* is the most widely used in the original prescription, which is the monarch drug in BHD. A systematic review and meta-analysis of nineteen RCTs with 1580 individuals showed that BHD could significantly improve the neurological deficit score and the ability of self-care of patients with IS [7]. BHD could promote angiogenesis, attenuate infiltration of natural killer cells, and facilitate neurorehabilitation through an improvement of synaptic plasticity after cerebral ischemia/reperfusion injury. It could significantly decrease cerebral edema and rat neurological function scores and reduce brain infarct volume [8–12]. Pharmacological researches have shown that *Radix Astragali* has the function of dilating blood vessels and improving microcirculation, which can significantly increase the brain's ability to withstand periods of severe hypoxia and/or ischemia [13, 14]. *Carthami flos*, *Persicae Semen*, *Radix Paeoniae Rubra*, *Angelicae sinensis Radix*, and *Chuanxiong Rhizoma* can effectively improve the microcirculation of the body, significantly inhibit the proliferation of fibrous tissue, and reduce the inflammatory response [15–17].

**Figure 1:**
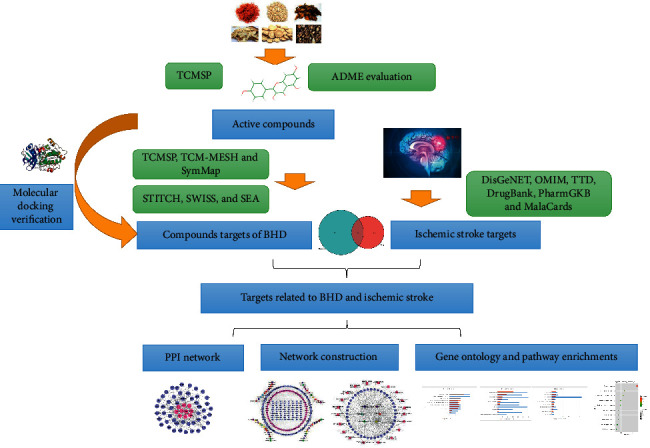
The flowchart of the whole study design.

However, the molecular mechanism of BHD has not been certainly clear. The clinical effect of the decoction is a comprehensive result of the complex biological processes in human body. Network pharmacology, based on system biology and multidirectional pharmacology, integrates the contents of computer biology and network analysis [18]. It explains the integrity and systematization of drug-target-disease interaction from the perspective of multicomponents, multitargets, and multichannels, which is consistent with the holistic view of Chinese medicine [19]. Therefore, it provides a method for the study of multicomponents' mechanisms of Chinese medicine [20, 21]. In this paper, network pharmacology was used to explore the molecular mechanism of BHD against IS. The detailed workflow of the study is shown in Figure 1.

## 2. Materials and Methods

### 2.1. Chemical Ingredients Collection and Active Compounds Screening

Traditional Chinese Medicine Systems Pharmacology Database (TCMSP, http://lsp.nwsuaf.edu.cn) [22], BATMAN-TCM (http://bionet.ncpsb.org/batman-tcm/index.php/Home/Index/index) [23], ETCM (http://www.tcmip.cn/ETCM/index.php/Home/index/index.html), and SymMap (https://www.symmap.org/) [24] were used to collect the compounds of BHD. Next, ADME analysis was carried out by collecting the main components of BHD according to the condition parameters (OB ≥ 30%, DL ≥ 0.18, Caco-2>−0.4) [25]. Through ADME analysis, the potential active components were screened out for further analysis.

### 2.2. Target Identification

TCMSP, BATMAN-TCM, ETCM, and SymMap databases were used to screen the targets of all the components of BHD. If related targets of components in BHD could not be found from the above databases, further target prediction was carried out in the STITCH (http://stitch.embl.de/) [26], SwissTargetPrediction (SwissTargetPrediction, http://www.Swisstargetprediction.ch/) [27], and Similarity Ensemble Approach (SEA, http://sea.bkslab.org/) [28] databases.

Targets related to IS were derived from six public databases, including DisGeNET (http://www.disgenet.org/) [29], OMIM (https://omim.org/) [28], TTD (http://bidd.nus.edu.sg/group/cjttd/), DrugBank (https://www.drugbank.ca/), PharmGKB (https://www.pharmgkb.org/), and MalaCards (https://www.malacards.org/) [30], with keywords “ischemic stroke.”

The targets were normalized to the official gene symbols using UniProt database (https://www.uniprot.org/) [31] with the species limited to “*Homo sapiens*.” Finally, the intersection targets of BHD active components and IS were obtained and drawn using a Venn diagram.

### 2.3. Protein-Protein Interaction Data

String 11.0 (https://string-db.org/) [32] is a database for storing known and predicted protein interactions, including direct and indirect protein interactions. It scores each protein interaction. A higher score means a higher confidence of protein interaction.

The selected intersection targets were imported into String for protein interaction analysis, and the protein interaction network was obtained with the species limited to “*Homo sapiens*” and a confidence score >0.7. The protein interaction data were imported into Cytoscape 3.6.0 (https://cytoscape.org/) to construct the PPI network.

### 2.4. Gene Ontology (GO) and Pathway Enrichment

DAVID (https://david.ncifcrf.gov/) [33] database integrates various types of database resources and uses the improved Fisher precision test algorithm to analyze the enrichment of gene sets. A cutoff *P* value and false discovery rate (FDR) < 0.05 were used to indicate statistical significance. GO annotation and KEGG PATHWAY analysis were carried out for the intersection genes. Finally, we could get the pathway maps from KEGG PATHWAY Database (https://www.kegg.jp/) [34].

### 2.5. Network Construction and Cluster

#### 2.5.1. Network Construction

Network construction was performed as follows: (1) BHD-IS PPI network; (2) compound-target network; (3) herb-target-pathway network.

All networks can be constructed via utilizing the network visualization software Cytoscape, which displays network graphically. Cytoscape makes it possible for data integration, analysis, and visualization of complicated networks. In the network diagram, “node” represents the active component and target in BHD, and “edge” represents the relationship between the active component and target. The “degree” parameter, presenting the number of connections between the nodes in the network, was used to evaluate important targets [30].

#### 2.5.2. Cluster of BHD-IS PPI Network

The closely related regions in protein-protein interaction networks are defined as topological modules or clusters. These clusters or functional modules can put nodes of similar or related function together in the same network. By the help of MCODE, a plug-in of Cytoscape, we can get clusters.

### 2.6. Molecular Docking Verification

To validate the compound-target associations, the AutoDock software (version 4.2) was used to perform the molecular docking program [35]. RCSB PDB (http://www.rcsb.org/) [36] was used to retrieve and download the 3D structure files of key target proteins. 3D structure files of compounds were downloaded from PubChem (https://pubchem.ncbi.nlm.nih.gov/) [37]. Finally, the AutoDock platform was used for molecular docking verification [38]. The binding energy was calculated to evaluate binding interactions between the compounds and their targets. The binding energy less than “−5” indicates a good binding interaction between the compound and target [39].

## 3. Results

### 3.1. Active Compounds

775 compounds were ultimately reserved: 87 in *Radix Astragali*, 189 in *Chuanxiong Rhizoma*, 119 in *Radix Paeoniae Rubra*, 125 in *Angelicae sinensis Radix*, 66 in *Persicae Semen*, 189 in *Carthami flo*s, and 4 in *Pheretima*. After ADME screening, 78 potential compounds (OB ≥ 30%, DL ≥ 0.18, Caco-2 ≥ 0.4) of seven herbal medicines in BHD were identified, including 16 from *Radix Astragali*, 6 from *Chuanxiong Rhizoma*, 20 from *Radix Paeoniae Rubra*, 2 from *Angelicae sinensis Radix*, 13 from *Persicae Semen*, 21 from *Carthami flos*, and 0 from *Pheretima*. The details of candidate compounds are described in Table 1. *Radix Astragali*, *Chuanxiong Rhizoma*, *Radix Paeoniae Rubra*, *Angelicae sinensis Radix*, *Persicae Semen*, *and Carthami flos* are simplified as RA, CR, RPR, ASR, PS, and CF, respectively.

### 3.2. Targets of BHD and IS

As 19 compounds of BHD had no targets in TCMSP, BATMAN-TCM, ETCM, and SymMap, Canonical SMILES of these compounds were found in PubChem. Based on chemical structural similarity, we used databases like STITCH, SwissTargetPrediction, and SEA, to predict their targets. These compounds were excluded because of the targets score less than 50% eventually. In brief, 235 targets were adopted in this research.

By means of the six available resources, namely, DisGeNET, OMIM, TTD, DrugBank, PharmGKB and MalaCards databases, we obtained 460 IS-related targets.

**Figure 2:**
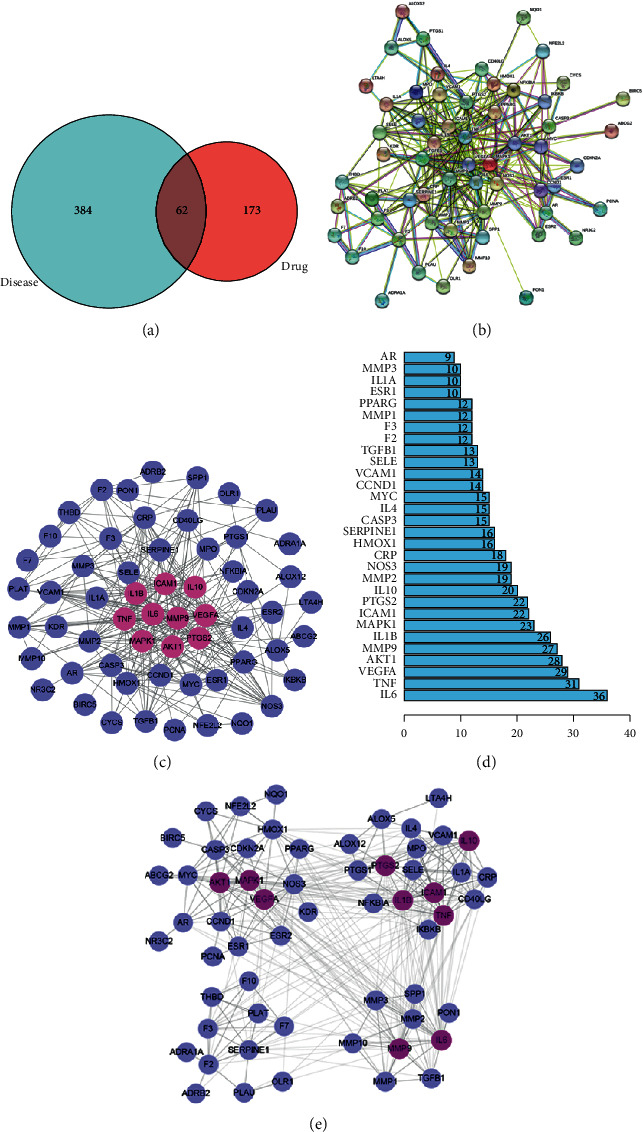
Topological analysis of the target proteins of BHD related to IS. (a) Venn diagram showing shared and unique targets of IS and BHD. (b) The protein-protein interaction (PPI) network diagram constructed by the String database. (c) BHD-IS PPI network constructed by Cytoscape. (d) Bar graph of all protein nodes degree related to the targets. (e) Cluster of PPI network. Lilac and rose red circles stand for 62 intersection targets, and the top ten targets are shown in rose red.

Based on targets of the candidate ingredients and IS, intersection targets were got by R software. 62 intersection genes were found eventually, shown in Figure 2(a). The details of intersection targets are described in Table 2.

### 3.3. Gene Ontology and Pathway Enrichment Analysis

#### 3.3.1. Gene Ontology

**Figure 3:**
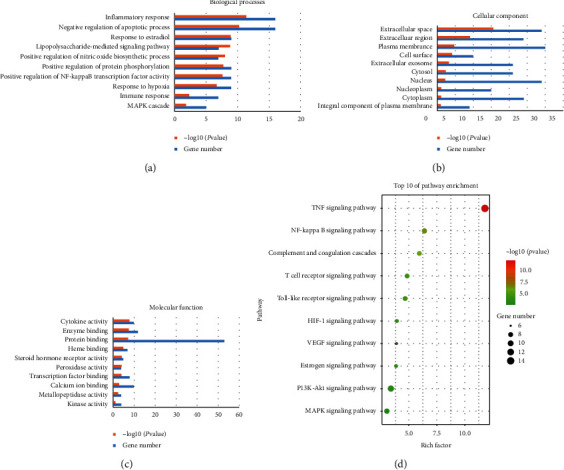
GO and KEGG PATHWAY enrichment analysis for targets of BHD related to IS. (a) Biological processes. (b) Cellular component. (c) Molecular function. (d) Bubble chart of KEGG PATHWAY analysis. The order of importance was ranked by −log10 (*P* value) and gene number.

GO analysis of 62 candidate targets for BHD against IS was performed using the DAVID database to understand the relationship between functional units and their underlying significance in the biological system networks. The results were divided into three parts including biological processes, cellular component, and molecular function, as shown in Figures 3(a)–3(c).

#### 3.3.2. Pathway Enrichment

Through comprehensive analysis, we obtained an integrated IS pathway based on our current knowledge of IS pathogenesis to illuminate the integral role of BHD in treating IS. TOP 10 KEGG signaling pathways of BHD were obtained and constructed based on *P* value as shown in Figure 3(d).

### 3.4. BHD-IS PPI Network Analysis

#### 3.4.1. BHD-IS PPI Network

62 intersection targets were imported into the String database, and TSV text showing the interaction relationship was obtained, as shown in Figure 2(b). Then, the network topology analysis was applied by the software of Cytoscape 3.6.0. Importing the TSV text into the Cytoscape software, we could get BHD-IS network, as shown in Figure 2(c). This network contained 59 nodes and 664 edges. In this network, the rose red nodes had higher degrees. The number of those nodes' edges was 36 in IL6, 31 in TNF, 29 in VEGFA, 28 in AKT1, 27 in MMP-9, 26 in IL1B, 23 in MAPK1, 22 in ICAM1, 22 in PTGS2, and 20 in IL10, respectively. This suggested that these genes might be the key or central genes in IS development. Bar graph of all protein nodes degree related to the targets is shown in Figure 2(d). The target proteins in the PPI network were modularized and analyzed by using the plug-in of cluster maker of the software Cytoscape 3.6.0. The results showed that 62 targets were divided into four modules, including 24 in module one, 18 in module two, 11 in module three, and 9 in module four, as shown in Figure 2(e). Functional annotation of the 4 modules is shown in Table 3.

#### 3.4.2. Compound-Target Network Analysis

**Figure 4:**
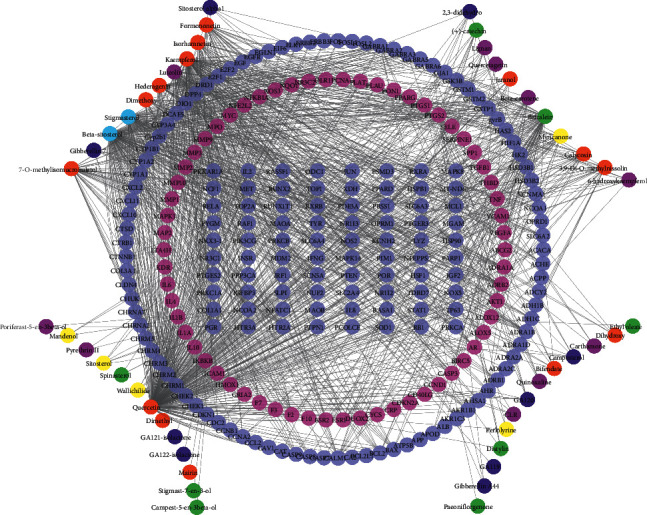
Compound-target network. Light purple and rose red circles stand for the total 235 compound targets, among which the light purple ones represent the 62 intersection targets of BHD and IS. The red, yellow, green, navy blue, light blue, and purple circles stand for active compounds of *Radix Astragali*, *Chuanxiong Rhizoma*, *Radix Paeoniae Rubra*, *Persicae Semen*, *Angelicae sinensis Radix*, and *Carthami flos*.

The active components and their targets were constructed to establish the compound-target network using Cytoscape. This network was composed of 294 nodes (235 compound-target nodes and 59 compound nodes) and 1192 edges. In this network, we could find that one target could be hit by several compounds (central nodes, such as IL6, MMP-9, TNF, AKT1, ICAM1, IL1B, PTGS2, IL-10, VEGFA, and MAPK1), but some were modulated by only one compound in this network. Furthermore, one potential active compound could correspond to multiple targets. Top twelve compounds with high degree were quercetin, beta-sitosterol, kaempferol, stigmasterol, baicalein, luteolin, hederagenin, 7-o-methylis omicron ulatol, formononetin, isorhamnetin, dimethoxy, and myricanone, shown in Table 4. It could be seen that the neuroprotective mechanism of BHD had the characteristics of multicomponents, multitargets, and multi mechanisms. The compound-target network is shown in Figure 4.

#### 3.4.3. Herb-Target-Pathway Network

**Figure 5:**
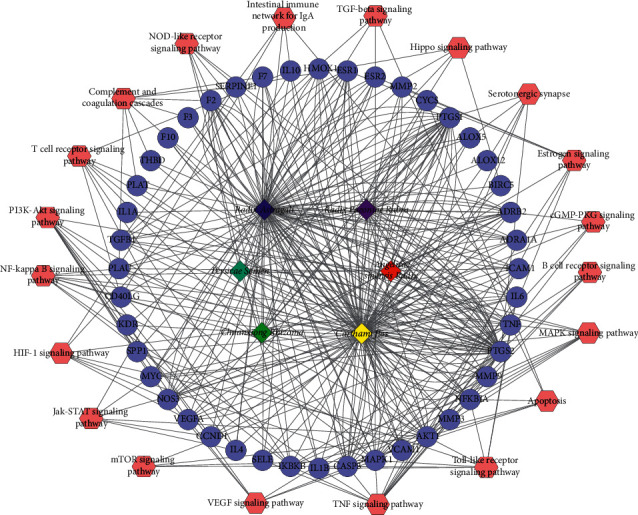
Herb-target-pathway network. The hexagons stand for 20 IS-related pathways and the red, yellow, green, navy blue, light blue, and purple quadrilateral stand for compounds of *Radix Astragali*, *Chuanxiong Rhizoma*, *Radix Paeoniae Rubra*, *Persicae Semen*, *Angelicae sinensis Radix*, and *Carthami flos*, respectively. Besides, lilac circles stand for genes related to BHD.

By importing 62 targets which overlapped with IS disease genes into DAVID, we could get 20 IS-related pathways. *Radix Astragali* and *Carthami flos* had the highest degree, which means that the two herbs might be the main herbs in treating IS. Meanwhile, TNF signaling pathway showed the highest degree, followed by PI3K-Akt signaling pathway, MAPK signaling pathway, NF-kappa B signaling pathway, Toll-like receptor signaling pathway, and T cell receptor signaling pathway, respectively. The herb-target-pathway network is shown in Figure 5.

### 3.5. Molecular Docking Verification

**Figure 6:**
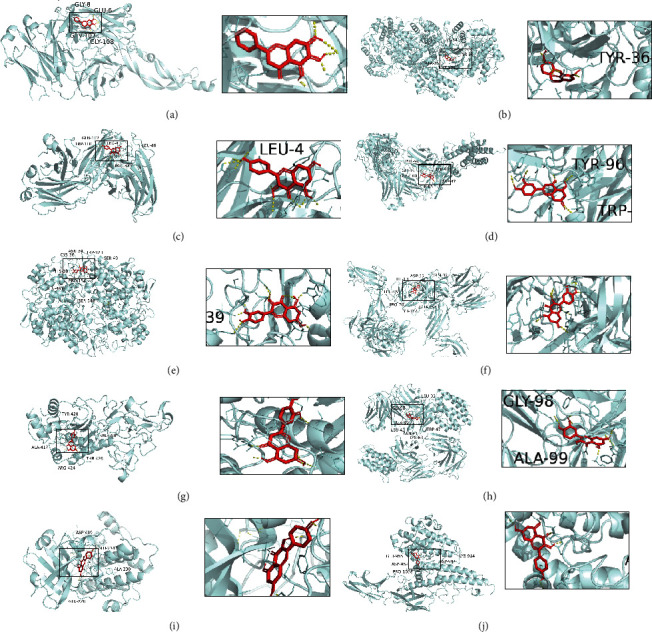
The conformations of some important compounds and key targets. (a) TNF with kaempferol (binding energy = −7.67). (b) PTGS2 with quercetin (binding energy = −9.29). (c) MMP-9 with luteolin (binding energy = −6.9). (d) IL6 with luteolin (binding energy = −8.12). (e) VEGFA with baicalein (binding energy = −6.67). (f) IL1B with quercetin (binding energy = −8.32). (g) ICAM1 with kaempferol (binding energy = −8.5). (h) MAPK1 with 7-O-methylisomucronulatol (binding energy = −6.7). (i) AKT1 with kaempferol (binding energy = −7.96). (j) IL-10 with quercetin (binding energy = −7.55).

Compound-target interactions with binding energy less than −5.0 kcal/mol are shown in Figure 6, including TNF with kaempferol (A), PTGS2 with quercetin (B), MMP-9 with luteolin (C), IL6 with luteolin (D), VEGFA with baicalein (E), IL1B with quercetin (F), ICAM1 with kaempferol (G), MAPK1 with 7-O-methylisomucronulatol (H), AKT1 with kaempferol (I), IL-10 with quercetin (J).

### 3.6. Target Path Analysis

**Figure 7:**
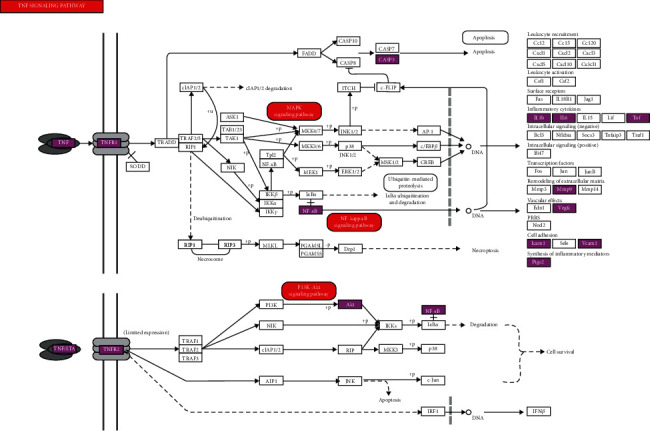
Pathway map of BHD against IS. The key targets of BHD in the treatment of IS are shown as rose red in the TNF signal pathway.

The pathway map of BHD in treating IS was obtained from KEGG PATHWAY Database, as shown in Figure 7. The related pathways were marked in red, and the targets of BHD in treating IS were marked in rose red. The results showed that the main pathways of BHD in treating IS included TNF signaling pathway, MAPK signaling pathway, NF-*κ*B signaling pathway, and PI3K/AKT signaling pathway.

## 4. Discussion

In our study, we found the molecular mechanism of BHD's neuroprotection effect against IS using network pharmacology strategy. The network pharmacology strategy is helpful to clarify the mechanism of TCM's function from a systematic viewpoint [40, 41]. Furthermore, this method provides a multidimensional research strategy for various complicated herbal decoctions. At present, the application of network pharmacology to study the mechanism of Chinese medicine has become a research hotspot. In this study, we found that 59 active components of BHD could act on 62 targets related to IS. Further analysis showed that BHD could act on many biological processes of IS and had an influence on the outcome of stroke through TNF, PI3K-Akt, MAPK, and NF-kappa B signaling pathway. It further confirmed that BHD had the characteristics of multicomponents, multichannels, and multitargets.

Core ingredients with the highest degree in compound-target network were considered to be responsible for neuroprotection, including quercetin, beta-Sitosterol, kaempferol, stigmasterol, baicalein, luteolin, hederagenin, 7-O-methylisomucronulatol, formononetin, isorhamnetin, Dimethoxy, and myricanone. Six of these components belong to *Radix Astragali* and *Carthami flos*. The results of network topology analysis showed that the degrees of *Radix Astragali* and *Carthami flos* were the highest in BHD. As the core herb in BHD, the dosage of *Radix Astragali* is the highest, indicating that the results of network pharmacology are consistent with the clinical application of Chinese medicine. Quercetin and kaempferol are common components of *Radix Astragali* and *Carthami flos*. It was found that quercetin could pass through the blood-brain barrier with the highest passage rate [42]. A research showed that quercetin had effects of antioxidant stress and promoting autophagy, which was helpful for the prevention and treatment of stroke [43, 44]. In addition, quercetin could also regulate protein phosphatase 2A subunit B (PP2A) to produce significant neuroprotective effects on rats with cerebral ischemia-reperfusion injury and HT22 cell model of glutamate injury [45]. Lu et al. found that quercetin could inhibit the expression and release of many inflammatory factors such as TNF-*α*, IL-1 *β*, and IL6 by reducing the production of NF–*κ*B in elderly mice [46]. Kaempferol, a common flavonoid compound, has been widely concerned because of its anti-inflammatory, antioxidant, antibacterial, and antiviral effects. The neuroprotective effect of kaempferol has been confirmed in the acute phase of cerebral infarction [47, 48]. One study confirmed that kaempferol inhibited oxygen-glucose deprivation (OGD) induced cell viability decline, oxidative stress, mitochondrial dysfunction, and apoptosis [49]. These findings suggested that kaempferol might be a promising choice for the intervention of IS. Baicalein is a common component of *Carthami flos* and *Radix Paeoniae Rubra*. As an important flavonoid compound, baicalein has many pharmacological effects, such as antioxidant stress, anti-inflammatory, antiexcitatory toxicity, antiapoptosis, stimulating neurogenesis, and promoting the expression of neuroprotective factors [50–52]. Liu et al. found that baicalein had protective effect on transient middle cerebral artery occlusion model rats and could significantly reduce the apoptosis of ischemic penumbra cells around the ischemic infarct of middle cerebral artery occlusion (MCAO) model rats [53]. As an ingredient of *Carthami flos*, luteolin could downregulate the expression of TLR4, TLR5, NF-*κ* B, and P-P38MAPK, upregulate the expression of p-ERK, and protect cerebral ischemia in rats [54]. Experiments performed in vivo also demonstrated that luteolin reduced the infarct volume. It was suggested that luteolin had a potential in the treatment of IS through inhibiting MMP-9 and activating PI3K/Akt signaling pathway [55]. Beta-sitosterol and stigmasterol are the common components of *Carthami flos*, *Persicae Semen*, *Radix Paeoniae Rubra*, and *Angelicae sinensis Radix*. They are both sterol compounds, mainly having the functions of reducing blood fat, antioxidation, and anti-inflammation [56].

PPI analysis showed that IL6, TNF, VEGFA, AKT1, MMP-9, IL1B, MAPK1, ICAM1, PTGS2, and IL10 were the top ten targets with high degrees. Followed by cluster of the PPI network, the network could be divided into four modules, which were mainly related to angiogenesis, inflammation, coagulation, and blood-brain barrier. Inflammation plays a critical role in the pathological process of stroke [57]. IL1B, IL10, TNF, IL6, and ICAM1 are closely related to the inflammatory response after stroke, among which IL-10 is an important anti-inflammatory factor, while L1B, TNF, and IL6 are proinflammatory factors. ICAM1 is an important adhesion molecule mediating the adhesion reaction, which plays an important role in stabilizing the interaction between cells and promoting the migration of leukocytes and endothelial cells. Ischemic cascade reaction leads to microglial activation, which will promote the release of proinflammatory cytokines (TNF-*α*, IL1B, and IL6) and anti-inflammatory cytokines (IL10 and TGF-*β*) [58]. MMP-9 is a kind of matrix metalloproteinases (MMPs) closely related to the development of IS, which promotes embryo development, inflammation, atherosclerosis, and other biological functions. Under the stimulation of cerebral ischemia and hypoxia, microglia and astrocytes produce part of MMP-9 under the guidance of inflammatory factors. By hydrolyzing the tight junction protein on the basement membrane of cerebrovascular, the integrity of blood-brain barrier is destroyed [59]. VEGF is a double-edged sword in the development of cerebral infarction. In the hyper acute stage of cerebral infarction, the increase of VEGF concentration increases the permeability of blood-brain barrier, leading to brain edema and aggravate clinical symptoms. In the postinfarction recovery stage, the high content of VEGF is conducive to the establishment of collateral circulation of ischemic focus and penumbra and the damage and repair of neurons [60]. AKT1 is one of serine/threonine-protein kinases (AKT1, AKT2, and AKT3), and it regulates many processes including metabolism, proliferation, cell survival, growth, and angiogenesis. AKT1 gene deletion induces dysfunction of vascular endothelial cells, migration, and survival of vascular smooth muscle cells [61].

Pathway enrichment analysis results showed that TNF signaling pathway, PI3K-Akt signaling pathway, MAPK signaling pathway, and NF-kappa B signaling pathway were the main pathways. TNF signaling pathway is an important inflammatory pathway. As an important cytokine, TNF can induce apoptosis, cell survival, inflammation, immunity, and other intracellular signaling pathways. TNFR1 signal transduction can induce the activation of many genes, which are mainly controlled by NF-kappa B and MAPK cascade. In this present study, TNF, IL-1B, MYC, and TGFB1 are potential targets of BHD, suggesting that BHD plays a neuroprotective role against ischemia-reperfusion injury through TNF signaling pathway. PI3K/Akt signaling pathway is one of the important pathways of cerebral ischemia and neuronal apoptosis. A study found that activating PI3K/Akt signal pathway could inhibit the apoptosis of nerve and reduce the occurrence of blood-borne brain edema. A series of studies have shown that many Chinese herbal extracts play a protective role in IS through this pathway [62, 63]. Another study found that baicalein also decreased the LC3-II/LC3-I ratio and promoted phosphorylation of the PI3K/Akt/mTOR signaling pathway which implied inhibition of autophagy. The reduction of phosphorylation Akt and glycogen synthase kinase-3beta (GSK3beta) induced by OGD was restored by Baicalein, which was associated with preserved levels of phosphorylation of PTEN [53, 64]. It was reported that baicalein could activate PI3K/AKT pathway, inhibit caspase activation, and reduce cerebral infarct volume in MCAO rats [65]. Besides, formononetin mediated neuroprotection against cerebral ischemia/reperfusion in rats via downregulation of the Bax/Bcl-2 ratio and upregulation PI3K/Akt signaling pathway [66]. The MAPK signaling pathway may be a therapeutic pathway for stroke [67]. Researches showed that suppressing the NF-*κ*B and MAPK signaling pathways would downregulate the expression of proinflammatory factors. The MAPK signaling pathway could be a promising candidate for future applications in CNS injury treatment [68]. BHD alleviated pressure overload induced cardiac remodeling by suppressing TGF-*β*/Smads and MAPKs signaling activated fibrosis [69].

However, our research also has some limitations. For example, the accuracy and integrity of existing databases need further verification. Higher quality databases of traditional Chinese medicine and more accurate background network databases are needed. Moreover, the results of network pharmacology need experimental support. The application of network pharmacology in the study of Chinese medicine is just in its start-up step. We need to promote the interdisciplinary researches integrating network science, bioinformatics, computer science, mathematics, and pharmacology in the future.

## 5. Conclusion

In this study, we explored and discussed the characteristic of “multicomponents, multitargets, and multichannels” of BHD-mediated IS treatment through the method of network pharmacology and molecular docking. In the future, we should provide experimental evidence for the neuroprotective effect of BHD against IS according to the results of network pharmacology research.

## Figures and Tables

**Table 1:** Active compounds of BHD and their parameters.

Herb	Mol ID	Molecule name	OB (%)	Caco-2	DL
RA	MOL000211	Mairin	55.38	0.73	0.78
RA	MOL000239	Jaranol	50.83	0.61	0.29
RA	MOL000296	Hederagenin	36.91	1.32	0.75
RA	MOL000033	(3S,8S,9S,10R,13R,14S,17R)-10,13-Dimethyl-17-[(2R,5S)-5-propan-2-yloctan-2-yl]-2,3,4,7,8,9,11,12,14,15,16,17-dodecahydro-1H-cyclopenta[a]phenanthren-3-ol	36.23	1.45	0.78
RA	MOL000354	Isorhamnetin	49.6	0.31	0.31
RA	MOL000371	3,9-Di-O-methylnissolin	53.74	1.18	0.48
RA	MOL000378	7-O-Methylisomucronulatol	74.69	1.08	0.3
RA	MOL000380	(6aR,11aR)-9,10-Dimethoxy-6a,11a-dihydro-6H-benzofurano [3,2-c] chromen-3-ol	64.26	0.93	0.42
RA	MOL000387	Bifendate	31.1	0.15	0.67
RA	MOL000392	Formononetin	69.67	0.78	0.21
RA	MOL000398	Isoflavanone	109.99	0.53	0.3
RA	MOL000417	Calycosin	47.75	0.52	0.24
RA	MOL000422	Kaempferol	41.88	0.26	0.24
RA	MOL000438	(3R)-3-(2-Hydroxy-3,4-dimethoxyphenyl)chroman-7-ol	67.67	0.96	0.26
RA	MOL000442	1,7-Dihydroxy-3,9-dimethoxy pterocarpene	39.05	0.89	0.48
RA	MOL000098	Quercetin	46.43	0.05	0.28
CR	MOL001494	Mandenol	42	1.46	0.19
CR	MOL002135	Myricanone	40.6	0.67	0.51
CR	MOL002140	Perlolyrine	65.95	0.88	0.27
CR	MOL002151	Senkyunone	47.66	1.15	0.24
CR	MOL002157	Wallichilide	42.31	0.82	0.71
CR	MOL000359	Sitosterol	36.91	1.32	0.75
RPR	MOL001918	Paeoniflorgenone	87.59	−0.09	0.37
RPR	MOL001925	Paeoniflorin_qt	68.18	−0.34	0.4
RPR	MOL002714	Baicalein	33.52	0.63	0.21
RPR	MOL000358	Beta-sitosterol	36.91	1.32	0.75
RPR	MOL000359	Sitosterol	36.91	1.32	0.75
RPR	MOL004355	Spinasterol	42.98	1.44	0.76
RPR	MOL000449	Stigmasterol	43.83	1.44	0.76
RPR	MOL000492	(+)-Catechin	54.83	−0.03	0.24
RPR	MOL006992	(2R,3R)-4-Methoxyl-distylin	59.98	0.17	0.3
RPR	MOL006994	1-o-Beta-d-glucopyranosyl-8-o-benzoylpaeonisuffrone_qt	36.01	−0.03	0.3
RPR	MOL006996	1-o-Beta-d-glucopyranosylpaeonisuffrone_qt	65.08	−0.05	0.35
RPR	MOL006999	Stigmast-7-en-3-ol	37.42	1.32	0.75
RPR	MOL007005	Albiflorin_qt	48.7	−0.38	0.33
RPR	MOL007008	4-Ethyl-paeoniflorin_qt	56.87	−0.17	0.44
RPR	MOL007012	4-o-Methyl-paeoniflorin_qt	56.7	0.4	0.43
RPR	MOL007016	Paeoniflorigenone	65.33	−0.13	0.37
RPR	MOL007018	9-Ethyl-neo-paeoniaflorin A_qt	64.42	−0.01	0.3
RPR	MOL007022	Evofolinb	64.74	0	0.22
RPR	MOL002883	Ethyl oleate (NF)	32.4	1.4	0.19
RPR	MOL005043	Campest-5-en-3beta-ol	37.58	1.32	0.71
ASR	MOL000358	Beta-sitosterol	36.91	1.32	0.75
ASR	MOL000449	Stigmasterol	43.83	1.44	0.76
PS	MOL001323	Sitosterol alpha1	43.28	1.41	0.78
PS	MOL001328	2,3-Didehydro GA70	63.29	−0.27	0.5
PS	MOL001339	GA119	76.36	−0.12	0.49
PS	MOL001340	GA120	84.85	0.38	0.45
PS	MOL001342	GA121-isolactone	72.7	−0.26	0.54
PS	MOL001343	GA122	64.79	−0.17	0.5
PS	MOL001344	GA122-isolactone	88.11	−0.18	0.54
PS	MOL001351	Gibberellin A44	101.61	−0.13	0.54
PS	MOL001358	Gibberellin 7	73.8	−0.18	0.5
PS	MOL001371	Populoside_qt	108.89	0.49	0.2
PS	MOL000296	Hederagenin	36.91	1.32	0.75
PS	MOL000358	Beta-sitosterol	36.91	1.32	0.75
PS	MOL000493	Campesterol	37.58	1.31	0.71
CF	MOL001771	Poriferast-5-en-3beta-ol	36.91	1.45	0.75
CF	MOL002680	Flavoxanthin	60.41	0.97	0.56
CF	MOL002694	4-[(E)-4-(3,5-Dimethoxy-4-oxo-1-cyclohexa-2,5-dienylidene)but-2-enylidene]-2,6-dimethoxycyclohexa-2,5-dien-1-one	48.47	0.81	0.36
CF	MOL002695	Lignan	43.32	0.42	0.65
CF	MOL002698	Lupeol-palmitate	33.98	1.52	0.32
CF	MOL002706	Phytoene	39.56	2.22	0.5
CF	MOL002707	Phytofluene	43.18	2.29	0.5
CF	MOL002710	Pyrethrin II	48.36	0.53	0.35
CF	MOL002712	6-Hydroxykaempferol	62.13	0.16	0.27
CF	MOL002714	Baicalein	33.52	0.63	0.21
CF	MOL002717	qt_Carthamone	51.03	−0.31	0.2
CF	MOL002719	6-Hydroxynaringenin	33.23	0.27	0.24
CF	MOL002721	Quercetagetin	45.01	−0.06	0.31
CF	MOL002757	7,8-Dimethyl-1H-pyrimido[5,6-g]quinoxaline-2,4-dione	45.75	0.06	0.19
CF	MOL002773	Beta-carotene	37.18	2.25	0.58
CF	MOL000358	Beta-sitosterol	36.91	1.32	0.75
CF	MOL000422	Kaempferol	41.88	0.26	0.24
CF	MOL000449	Stigmasterol	43.83	1.44	0.76
CF	MOL000006	Luteolin	36.16	0.19	0.25
CF	MOL000953	CLR	37.87	1.43	0.68
CF	MOL000098	Quercetin	46.43	0.05	0.28

**Table 2:** Determined target information of BHD related to IS.

UniProt ID	Protein name	Gene name
Q9UNQ0	ABCG2	ATP-binding cassette sub-family G member 2
P35348	ADRA1A	Alpha-1A adrenergic receptor
P07550	ADRB2	Beta-2 adrenergic receptor
P31749	AKT1	RAC-alpha serine/threonine-protein kinase
P18054	ALOX12	Arachidonate 12-lipoxygenase, 12S-type
P09917	ALOX5	Arachidonate 5-lipoxygenase
P10275	AR	Androgen receptor
O15392	BIRC5	Baculoviral IAP repeat-containing protein 5
P42574	CASP3	Caspase-3
P24385	CCND1	G1/S-specific cyclin-D1
P29965	CD40LG	CD40 ligand
P42771	CDKN2A	Cyclin-dependent kinase inhibitor 2A
P02741	CRP	C-reactive protein
P99999	CYCS	Cytochrome c
Q9NRD8	DUOX2	Dual oxidase 2
P03372	ESR1	Estrogen receptor 1
Q92731	ESR2	Estrogen receptor 2
P00742	F10	Coagulation factor Xa
P00734	F2	Thrombin
P13726	F3	Tissue factor
P08709	F7	Coagulation factor VII
P42262	GRIA2	Glutamate receptor 2
P09601	HMOX1	Heme oxygenase 1
P05362	ICAM1	Intercellular adhesion molecule 1
O14920	IKBKB	Inhibitor of nuclear factor kappa-B kinase subunit beta
P22301	IL10	Interleukin-10
P01583	IL1A	Interleukin-1A
P01584	IL1B	Interleukin-1B
P05112	IL4	Interleukin-4
P05231	IL6	Interleukin-6
P35968	KDR	Vascular endothelial growth factor receptor 2
P09960	LTA4H	Leukotriene A-4 hydrolase
P11137	MAP2	Microtubule-associated protein 2
P28482	MAPK1	Mitogen-activated protein kinase 1
P03956	MMP-1	Matrix metalloproteinase-1
P09238	MMP-10	Matrix metalloproteinase-10
P08253	MMP-2	Matrix metalloproteinase-2
P08254	MMP-3	Matrix metalloproteinase-3
P14780	MMP-9	Matrix metalloproteinase-9
P05164	MPO	Myeloperoxidase
P01106	MYC	Myc proto-oncogene protein
Q16236	NFE2L2	Nuclear factor erythroid 2-related factor 2
P25963	NFKBIA	NF-kappa-B inhibitor alpha
P29474	NOS3	Nitric oxide synthase, endothelial
P15559	NQO1	NAD(P)H dehydrogenase [quinone] 1
P08235	NR3C2	Mineralocorticoid receptor
P78380	OLR1	Oxidized low-density lipoprotein receptor 1
P12004	PCNA	Proliferating cell nuclear antigen
P00750	PLAT	Tissue-type plasminogen activator
P00749	PLAU	Urokinase-type plasminogen activator
P27169	PON1	Serum paraoxonase/arylesterase 1
P37231	PPARG	Peroxisome proliferator activated receptor gamma
P23219	PTGS1	Prostaglandin G/H synthase 1
P35354	PTGS2	Prostaglandin G/H synthase 2
P16581	SELE	E-selectin
P05121	SERPINE1	Plasminogen activator inhibitor 1
P10451	SPP1	Osteopontin
P01137	TGFB1	Transforming growth factor beta-1
P07204	THBD	Thrombomodulin
P01375	TNF	Tumor necrosis factor
P19320	VCAM1	Vascular cell adhesion protein 1
P15692	VEGFA	Vascular endothelial growth factor A

**Table 3:** The main GO enrichment analysis terms of cluster of PPI network.

Cluster	Term	Genes	*P* value	FDR
1	GO:0045766 positive regulation of angiogenesis	NOS3, KDR, HMOX1, NFE2L2, VEGFA	9.29*E* − 06	0.001148
2	GO:0006954∼inflammatory response	IL10, CRP, IKBKB, IL1A, CD40LG, IL1B, PTGS2, SELE, TNF, PTGS1	2.87*E* − 11	1.10*E* − 08
3	GO:0007596∼blood coagulation	THBD, F7, F10, PLAU, PLAT, F2, F3	3.23*E* − 10	2.95*E* − 08
4	GO:0022617∼extracellular matrix disassembly	MMP-1, MMP-2, MMP-3, SPP1, MMP-9, MMP-10	9.21*E* − 11	2.95*E* − 08

**Table 4:** The top 12 active components in BHD.

Component	Traditional Chinese medicine	Degree	Betweenness centrality
Quercetin	*Carthami flos*	300	0.5468777

Beta-sitosterol	*Radix Paeoniae Rubra*	144	0.06546218
	*Angelicae sinensis Radix*		
	*Persicae Semen*		
	*Carthami flos*		

Kaempferol	*Hedysarum multijugum Maxim.*	124	0.12441394
	*Carthami flos*		

Stigmasterol	*Radix Paeoniae Rubra*	87	0.05677811
	*Angelicae sinensis Radix*		
	*Carthami flos*		

Baicalein	*Radix Paeoniae Rubra*	70	0.07380744
	*Carthami flos*		

Luteolin	*Carthami flos*	54	0.10769597

Hederagenin	*Hedysarum multijugum Maxim.*	44	0.03690132
	*Persicae Semen*		

7-O-Methylisomucronulatol	*Hedysarum multijugum Maxim.*	43	0.07061395
Formononetin	*Hedysarum multijugum Maxim.*	36	0.06178718
Isorhamnetin	*Hedysarum multijugum Maxim.*	34	0.04253939
Dimethoxy	*Hedysarum multijugum Maxim.*	25	0.01472432
Myricanone	*Chuanxiong Rhizoma*	23	0.01451958

## Data Availability

All data obtained or analyzed during this study are included within the article.
